# Viral Load as a Factor Affecting the Fatality of Patients Suffering from Severe Fever with Thrombocytopenia Syndrome

**DOI:** 10.3390/v14050881

**Published:** 2022-04-23

**Authors:** Heyon-Na Jo, Jieun Kim, Seong-Yeon Hwang, Jun-Won Seo, Da Young Kim, Na-Ra Yun, Dong-Min Kim, Choon-Mee Kim, Sook In Jung, Uh Jin Kim, Seong Eun Kim, Hyunah Kim, Eu Suk Kim, Jian Hur, Young Keun Kim, Hye Won Jeong, Jung Yeon Heo, Dong Sik Jung, Hyungdon Lee, Sun Hee Park, Yee Gyung Kwak, Sujin Lee, Seungjin Lim

**Affiliations:** 1 Department of Internal Medicine, College of Medicine, Chosun University, 588 Seosuk-dong, Dong-gu, Gwangju 61452, Korea; grangbleu@hanmail.net (H.-N.J.); hsokr@nate.com (S.-Y.H.); kaist-105@daum.net (J.-W.S.); dayz02@hanmail.net (D.Y.K.); shine@chosun.ac.kr (N.-R.Y.); 2 Department of Internal Medicine, College of Medicine, Hanyang University, Seoul 04763, Korea; quidam76@hanyang.ac.kr; 3 Premedical Science, Chosun University College of Medicine, Gwangju 61452, Korea; choonmeekim@chosun.ac.kr; 4 Department of Internal Medicine, Chonnam National University Medical School, Gwangju 61469, Korea; sijung@chonnam.ac.kr (S.I.J.); astralio@naver.com (U.J.K.); favorofgod@hanmail.net (S.E.K.); 5 Division of Infectious Diseases, Keimyung University Dongsan Medical Center, Daegu 42601, Korea; hyunah1118@dsmc.or.kr; 6 Department of Internal Medicine, Seoul National University Bundang Hospital, Seoul National University College of Medicine, Seongnam 13620, Korea; eskim@snubh.org; 7 Department of Internal Medicine, Yeungnam University Medical Center, Daegu 41158, Korea; sarang7529@yu.ac.kr; 8 Department of Internal Medicine, Wonju College of Medicine, Yonsei University Wonju, Wonju 26426, Korea; amoxj@yonsei.ac.kr; 9 Department of Internal Medicine, College of Medicine, Chungbuk National University, Cheongju 28644, Korea; hwjeong@chungbuk.ac.kr; 10 Department of Infectious Diseases, School of Medicine, Ajou University, Suwon 16499, Korea; jyeon78@naver.com; 11 Department of Internal Medicine, College of Medicine, Dong-A University, Busan 49315, Korea; dsjung@dau.ac.kr; 12 Department of Internal Medicine, College of Medicine, Hallym University, Chuncheon 24252, Korea; easydr@hallym.or.kr; 13 Division of Infectious Diseases, Department of Internal Medicine, College of Medicine, The Catholic University of Korea, Seoul 03083, Korea; sh.park@catholic.ac.kr; 14 Department of Internal Medicine, Inje University Ilsan Paik Hospital, Goyang 10380, Korea; ygkwak@paik.ac.kr; 15 Department of Internal Medicine, College of Medicine, Pusan National University, Yangsan 50612, Korea; beauty192@hanmail.net (S.L.); babopm@naver.com (S.L.)

**Keywords:** SFTS phlebovirus, viral RNA load, mortality

## Abstract

The clinical characteristics and the effect of viral RNA loads on fatality in 56 patients with severe fever with thrombocytopenia syndrome (SFTS) were analyzed. The non-survival group (12 patients) demonstrated a significantly higher mean age (77 years) than the survival group (44 patients, 65 years) (*p* = 0.003). The survival rates were 91.7% and 8.3% in patients with Ct values ≥30 and differed significantly (*p* = 0.001) in the survival and non-survival groups, respectively. The survival rates were 52.4% and 47.6% in patients with viral copy numbers ≥10,000 and 94.3% and 5.7% in patients with viral copy numbers <10,000 in the survival and non-survival groups, respectively (*p* = 0.001). In a multivariate analysis, viral copy numbers and initial Acute Psychologic Assessment and Chronic Health Evaluation II (APACHE II) scores were identified as the factors affecting fatality (*p* = 0.015 and 0.011, respectively). SFTS viral RNA loads can be useful markers for the clinical prediction of mortality and survival.

## 1. Introduction

Severe fever with thrombocytopenia syndrome (SFTS) is a tick-borne viral zoonotic disease that has been reported throughout China, Korea, and Japan since 2009 [1]. SFTS is characterized by acute fever, thrombocytopenia, leukopenia, elevated levels of liver enzymes, gastrointestinal symptoms, and multiorgan failure [2]. Cases of SFTS infection have recently been observed in Vietnam and Taiwan [2,3]. SFTS virus (SFTSV) is an RNA virus of the family *Bunyaviridae*, genus *Phlebovirus* [4]. The Korea Center for Disease Control and Prevention reported that the number of patients with SFTS in South Korea has been increasing every year since the first confirmed case in 2013 and reached 607 in 2017, with a survival rate of 21% (127 deaths) [5].

According to a study analyzing the relationship between the number of SFTSV RNA copies established via real-time PCR and fatality in patients with SFTS, the non-survivors had lower Ct values and a higher number of viral RNA copies compared to the SFTS survivors [6]. Research examining the relationship between the changes in viral RNA loads of SFTSV, clinical symptoms of SFTS patients, and fatality due to SFTS is currently lacking. Hence, this study aimed to compare the Ct values, the number of SFTSV copies, and the clinical characteristics between the survivors and non-survivors.

## 2. Methods

### 2.1. Data Collection

The confirmed cases of SFTS were selected from suspected cases who fulfilled one or both of the following criteria: (1) detection of SFTSV RNA via at least two variants of nested PCRs targeting different SFTSV segments (M or S); (2) SFTSV isolation. A total of 59 patients from 9 institutions were registered in the study with the approval of the Institutional Review Board. After receiving approval from Institutional Review Boards (Yeungnam University Hospital, Ajou University Hospital, Chungbuk University Hospital, Chonnam national University Hospital, Wonju Severance Christian Hospital, Dong-A University Hospital, Keimyung University Hospital, Hanyang University Hospital, and Chosun University Hospital), written formal consent was obtained from the patients.

Three patients were excluded due to insufficient medical records. Patients demonstrating co-infections were also excluded from this study. The research examined patients aged 19 years and older diagnosed with SFTS between 2015 and 2018.

### 2.2. Nucleic acid Extraction and cDNA Synthesis

Whole blood samples were collected from the SFTS patients. The average sample collection date (sample collection date after symptom occurrence) within 7 days was 5.19 days, and the average sample collection date (sample collection date after symptom occurrence) after 7 days was 11.5 days. Viral RNA was extracted from 300 µL blood samples using the Viral Gene-spinRNA Extraction Kit (iNtRON Biotechnology, Seongnam, Korea) following the manufacturer’s instructions. The extracted RNA was stored at −70 °C before use. The cDNA was synthesized using the SuperScriptVILO MasterMix (Thermo Fisher, San Francisco, CA, USA) following the manufacturer’s instructions. The synthesized cDNA was stored at −20 °C until further use.

### 2.3. Primers and Probes for Real-Time PCR

The SFTSV nested PCR targeting the M-segment (n-PCR-M) was performed using an inner primer [SFTS-F(MF3)/SFTS-R(MF2)] from a previously published report [7], whereas we designed the outer primer set SFTS-M 1st-F (TCATCCTGACTATTYTAGCAATWG) and SFTS-M 1st-R (TAAGTYACACTCACACCCTTGAA) for the first round of PCR.

Nested PCR targeting the S-segment (n-PCR-S) of SFTSV was performed using primers SFTS-S-NP-2F/SFTS-S-NP-2R and SFTS-S-N2F/SFTS-S-N2R [8]. PCR was performed using a 20 µL sample containing 2 µL of cDNA template, 0.5 pmol of specific primers, and the AccuPower Taq PCR PreMix (Bioneer, Daejeon, Korea).

For real-time PCR, the SFTS-SQ-F/SFTS-SQ-R/SFTS-SQ-P developed by Zhang et al. was used to amplify the S-segment of the SFTSV [9]. The information of the primers and probes are shown in Supplementary Table S1.

PCR was performed using a 20 µL sample containing 2 µL of cDNA template, 0.5 pmol of specific primers, 0.25 pmol of specific probe, and 4 µL of the LightCycler TaqMan Master mix. Amplification and detection were performed with an Exicycler Quantitative Thermal Block (Bioneer, Daejeon, Korea) under the following conditions: 5 min of pre-denaturation at 95 °C, 45 cycles of 5 s of denaturation at 95 °C, and 5 s of primer annealing at 55 °C.

Positive control plasmids were synthesized as a reference to quantify SFTSV RNA. The plasmid concentration was measured using a NanoDrop spectrophotometer (Thermo Fisher, San Francisco, CA, USA). The number of SFTSV RNA copies was calculated using an online tool (http://cels.uri.edu/gsc/cndna.html accessed on 19 April 2022). Thereafter, the positive control plasmids were serially diluted from 10^8^ to 10^1^ and used in real-time PCR to obtain a calibration curve.

### 2.4. Statistical Analysis

Categorical variables were expressed as frequencies and ratios. Continuous variables were expressed as the mean with standard deviation (SD) and interquartile range (IQR). The chi-squared test or Fisher’s test were used to compare the non-survival and survival groups (categorical variables). Continuous variables were analyzed using Spearman’s rho test and nominal variables using the Mann–Whitney *U* test. Fisher’s exact test and Mann–Whitney *U* test were used to analyze clinical characteristics. All statistical analyses were performed using the SPSS version 22.0. The level of statistical significance was set at *p* < 0.05. Patients were divided into two groups based on the number of viral copies. Medcalc version 18 was used to compare the survival rates based on the Ct values and the number of copies of SFTSV RNA.

## 3. Results

Among 56 patients, 44 were classified as the survival group and 12 as the non-survival group. Each group was screened for the clinical characteristics. The signs and symptoms that are associated with SFTS were also considered for the screening of each group. All groups were screened for body temperature (>38 degree), thrombocytopenia (PLT < 100,000), leukocytosis (<4000), or whether patients identified with SFTSV. The non-survival group had a higher mean age (77 years) than the survival group (65 years) (*p* = 0.003) (Table 1).

Within 24 h after admission to an ICU, we calculated the Acute Psychologic Assessment and Chronic Health Evaluation II (APACHE II) scores, an integer score based on the severity-of-disease classification system. The non-survival group demonstrated a higher initial APACHE II score obtained after hospital admission (14 points) compared to that of the survival group (10 points) (*p* = 0.005). Additionally, a significant difference in the rate of ICU admission was observed between the two groups (*p* < 0.001). Twenty patients (45.5%) in the survival group and two patients (16.7%) in the non-survival group developed headaches. Eight patients (18.2%) in the survival group and seven patients (58.3%) in the non-survival group demonstrated changes in their mental health status. The differences in the rates of these clinical symptoms between the two groups were statistically significant (*p* = 0.008 and *p* = 0.020, respectively).

Patients were divided into two groups according to the number of SFTSV RNA copies. SFTSV RNA copy numbers were <10,000 in 35 patients (62.5%) and ≥10,000 in 21 (37.5%) patients. Table 2 summarizes the clinical characteristics of each group.

The fatality and ICU admissions were associated with viral RNA loads (*p* < 0.001 and *p* = 0.003, respectively). The non-survival group had a significantly higher viral RNA load than the survival group (*p* < 0.001) within seven days after the symptom onset, and it increased over time.

**Figure 1:**
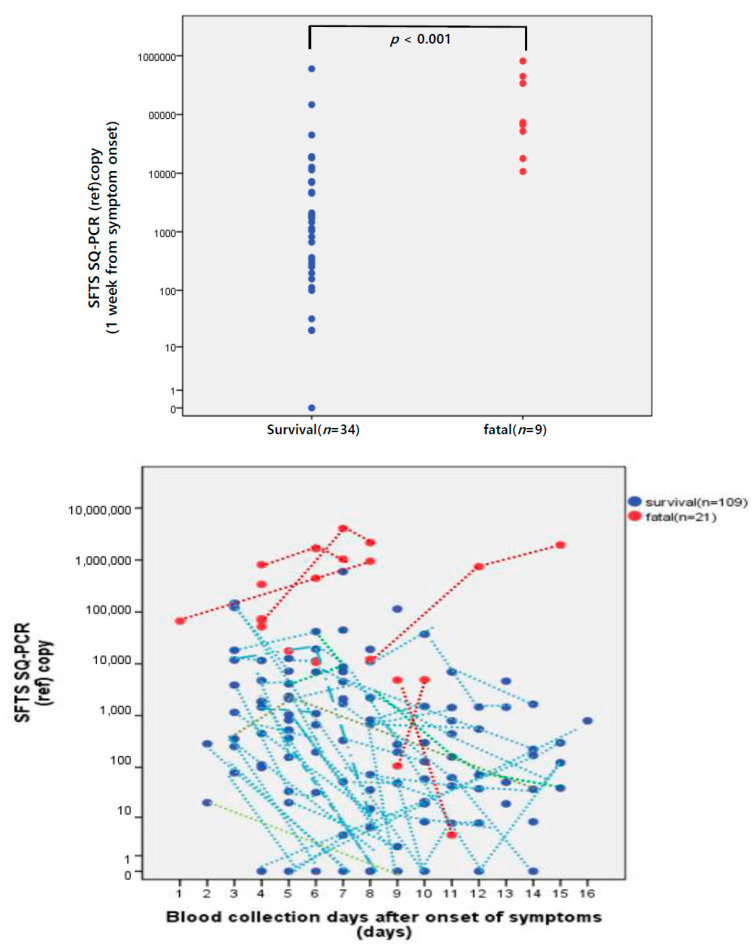

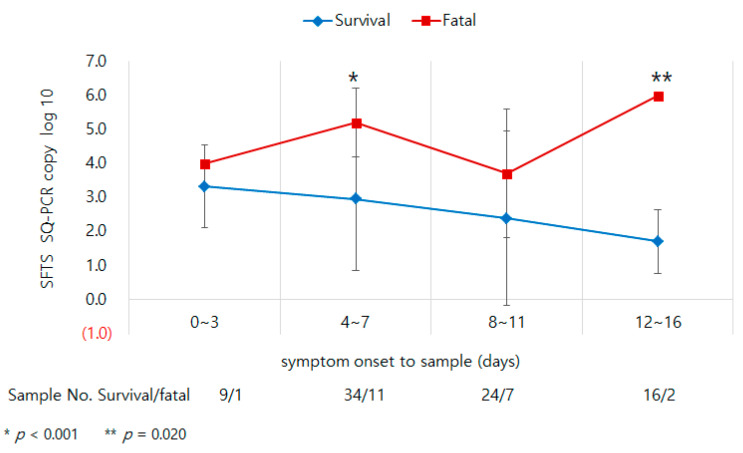
Comparison of SFTS SQ-PCR copy numbers between the survival group (*n*= 34) and the fatality group (*n* = 9) during the first week (days 0–7) from symptom onset to the time of sampling. Kinetics of SFTS viral copy numbers in blood specimens collected after symptom onset. Kinetics of the log_10_ SFTS SQ-PCR copy in the survival group and the non-survival group from symptom onset to the time of sampling. * *p* < 0.001, ** *p* = 0.020.

The number of viral RNA copies was maintained in the range of 10,000 into 1,000,000 copies/mL in the non-survival group. A significant difference was observed in the number of copies of viral RNA between the two groups (*p* < 0.001) in one week from the symptom onset (Figure 1).

The number of copies of viral RNA significantly decreased over time in the survival group (*p* = 0.001) (Figure 1).

Although the changes in the number of copies could not be fully tracked in the non-survival group, as a few patients died while receiving treatment, the non-survival group demonstrated a significantly higher viral RNA load of 5.1 log_10_ genome copies/mL than the survival group with a viral RNA load of 2.9 log_10_ genome copies/mL from days to 4–7 after the symptom onset (*p* < 0.001). The non-survival group demonstrated a significantly higher viral RNA load of 6.1 log_10_ genome copies/mL compared to the survival group with a viral RNA load of 2.1 log_10_ genome copies/mL from days to 12–16 after symptom onset (*p* = 0.020) (Figure 1).

Of the 21 cases with SFTSV copy numbers > 10,000, 11 (52.4%) survived and 12 (47.6%) were fatal. Of the 35 cases with SFTSV copy numbers < 10,000, 33 (94.3%) survived and two (5.7%) were fatal. The correlation diagram between the Ct value and the SFTS viral copy number showed the statistically significant difference in both the survival and non-survival group with the *p* values of 0.0496 and 0.0101, respectively.

**Figure 2:**
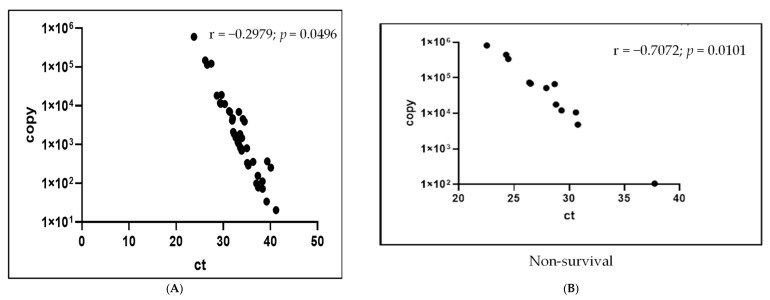
(**A**) Correlation diagram between the Ct value and the SFTS viral copy number of the survival group. (**B**) Correlation diagram between the Ct value and the SFTS viral copy number of the non-survival group.

The differences in the Ct values according to the number of SFTSV RNA copies were statistically significant (Figure 2A,B).

**Figure 3:**
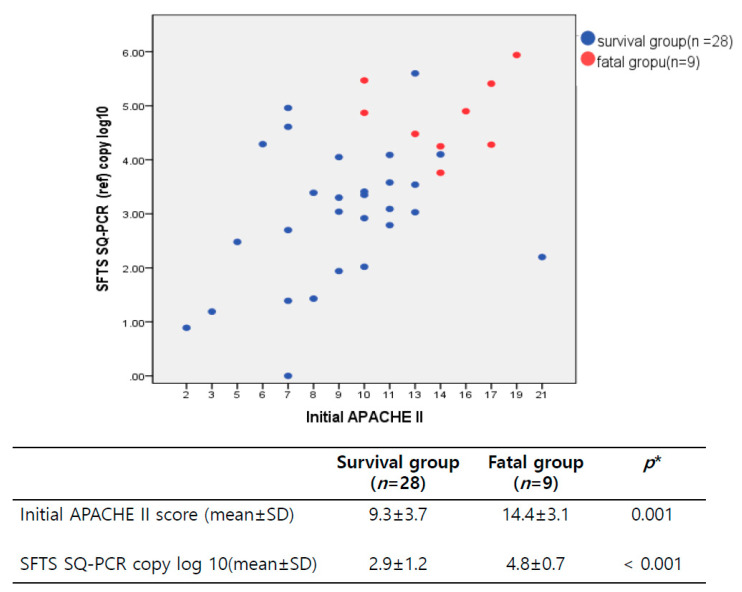
Correlation between the APACHE II score and the viral RNA load in SFTS patients 7 days from the symptom onset to the sample collection.

At the time of admission, the non-survival group demonstrated a higher APACHE II score of 14.4 ± 3.1 points than the survival group, with a score of 9.3 ± 3.7 points (*p* = 0.001) and a significantly higher SFTS viral RNA load of 4.8 log_10_ genome copies/mL than the 2.9 log_10_ genome copies/mL demonstrated by the survival group (*p* < 0.001) (Figure 3).

In a univariate analysis of mortality risk factors, the number of SFTSV RNA copies (cutoff 10,000), initial APACHE II scores, and ICU admission rates were significantly associated with fatality (*p* = 0.001, *p* = 0.012, and *p* = 0.001, respectively). In a multivariate analysis of the factors associated with mortality, only the number of SFTSV RNA copies (cutoff 10,000) and initial APACHE II scores were identified as the risk factors (*p* = 0.015, and *p* = 0.011, respectively) (Table 3).

## 4. Discussion

The number of SFTSV RNA copies has been reported to provide relevant information for prognosis prediction or treatment planning for patients with SFTS, along with cytokine, lactate dehydrogenase, aspartate aminotransferase, and blood urea nitrogen levels [8]. In a previous study examining the relationship between the number of SFTSV viral copies and fatality, a non-survival group demonstrated lower Ct values in real-time PCR and a higher number of viral copies compared to the survival group [10]. In another study, while a survival group and a non-survival group demonstrated high viral RNA loads from days 1–7 after the symptom onset, the viral RNA load decreased in the survival group and increased in the non-survival group from days to 7–13 after the symptom onset [11].

Yang et al. reported that the correlation increased between the viral RNA load and the laboratory hematological and biochemical parameters. However, only five fatal cases were included, and the statistical significance between the survival and non-survival group was not reported [6]. Kwon et al. also reported the correlation between patients with SFTS viremia and kinetics of cytokines in the plasma samples to investigate the pathogenesis of SFTS [12]. A total of 11 patients were enrolled in the study, including only one case of in-hospital mortality. Song et al. reported that high-level viremia, reduced platelets, coagulation dysfunction, multi-organ injuries, elevated IL-6, and TNF-α were closely associated with the aggravation of SFTS. They did not check viral kinetics during disease progression [13]. However, insufficient investigation regarding the correlation between fatality, statistical significance between survival and non-survival group, clinical symptoms, and fluctuating viral copy number of SFTS patient creates the necessity to demonstrate the applicability of the viral RNA load as a marker of SFTS in a clinical setting.

The results of this study delineated the usefulness of the number of viral copies measured with real-time PCR as a predictive factor for fatality in patients with SFTS. Furthermore, the research aided in determining the Ct value for SFTSV RNA and proposed a cutoff value for the number of viral copies as a fatality predictor.

APACHE II scores are a measure of disease severity that are evaluated based on clinical data and help in calculating the probability of death [14]. Studies have demonstrated the usefulness of APACHE II scores as a predictor of fatality in patients with severe community-acquired pneumonia [15] and acute kidney injury [16]. Consistent with the report that the fatality rate increases with an increase in APACHE II scores [14], in this study, the non-survival group demonstrated higher APACHE II scores than the survival group. In the multivariate analysis, the number of SFTSV copies and initial APACHE II scores were identified as fatality risk factors. While some studies have demonstrated that the fatality rate increases with viral RNA loads, few studies have investigated the accuracy of this claim. In this study, an SFTSV RNA copy number greater than or equal to 10,000 (collected at seven days post-symptom onset) and initial APACHE II scores were identified as fatality predictors. The number of SFTSV RNA copies and initial APACHE II scores may be used to predict fatality and prognoses within seven days after the symptom onset and aid in the planning of future treatment for patients with SFTS.

However, it is important to note that the limitations of this study include insufficient sample size, the difficulty of standardization of the quantification methods such as SFTS viral copies and Ct value, and the lack of follow-up tests of the blood samples of non-survivors. Additionally, in our study, we performed the multivariate analysis with five factors, but the n was too small to enroll in the analysis.

## 5. Conclusions

Based on the results of the multivariate analysis, the number of SFTSV RNA copies and initial APACHE II scores were identified as the risk factors of fatality in patients with SFTS. This study confirmed that viral RNA loads are useful markers that can predict fatality and survival in patients with SFTS in a clinical setting.

## Figures and Tables

**Table 1:** Clinical characteristics of patients with SFTS (*n* = 56).

Clinical Characteristics	Number of Patients (%)			
	Total (*n* = 56)	Non-Survival (*n* = 12)	Survival (*n* = 44)	*p*
Age, years (median, IQR)	66 (58–76)	77 (72–83)	65 (55–73)	0.003
Sex (No. of M/No. of F)	27 (93.1%)	4 (50%)	23 (85.2%)	0.249
Occupation				
Agriculture	22 (39.3%)	3 (14%)	19 (86.4%)	0.484
Tick bite				
Memory of tick bite	17 (30.4%)	4 (23.5%)	13 (76.5%)	0.826
Presence of bite wound	20 (35.7%)	5 (25%)	15 (75%)	0.122
APACHE II score upon admission	10 (8–13)	14 (10–17)	10 (7–13)	0.005
ICU admission	21	10 (57.1%)	11 (52.4%)	<0.001
Symptom onset to admission (median days, IQR)	4 (3–6)	5 (4–9)	5 (3–6)	0.538
Clinical manifestations				
Fever	53 (94.6%)	12 (100%)	41 (93.2%)	0.352
Chills	35 (62.5%)	5 (41.7%)	30 (68.2%)	0.188
Myalgia	27 (48.2%)	3 (25%)	24 (54.5%)	0.153
Arthralgia	8 (14.3%)	1 (8.3%)	7 (15.9%)	0.310
Nausea/vomiting	25 (44.6%)	5 (41.7%)	20 (45.5%)	0.815
Diarrhea	20 (35.7%)	3 (25%)	17 (38.6%)	0.126
Headache	22 (39.3%)	2 (16.7%)	20 (45.5%)	0.008
Rash	6 (10.7%)	1 (8.3%)	5 (11.4%)	0.825
Altered Mental state	15 (26.8%)	7 (58.3%)	8 (18.2%)	0.020
Underlying diseases	30 (53.6%)	9 (75%)	21 (47.7%)	0.108
Lab findings				
WBC (median, IQR)	N = 56 1895 (1325–2575)	N = 12 1755 (1300–2167)	N = 44 1990 (1325–2730)	0.641
Neutrophils (median, IQR)	N = 54 62.3 (53.7–70.3)	N = 12 70.3 (60.9–77.3)	N = 42 60.5 (52.8–68.4)	0.623
PLT (median, IQR)	N = 56 80.5 (56.8–99.8)	N = 12 60 (42–94)	N = 44 83.5 (59–105.8)	0.466
CRP (median, IQR)	N = 15 0.73 (0.06–1.51)	N = 4 1.26 (0.8–1.87)	N = 11 0.14 (0.03–0.88)	0.241
CK (median, IQR)	N = 37 541 (191–1151)	N = 8 464 (164–1087)	N = 29 541 (191–1329)	0.514
LDH (median, IQR)	N = 32 966 (629–1626)	N = 8 1230 (695–4327)	N = 24 899.5 (513–1380)	0.5
aPTT (median, IQR)	N = 48 39.5 (34.9–47.7)	N = 10 46.8 (36.4–59.5)	N = 38 38.3 (34.3–46.3)	0.389
Bilirubin (median, IQR)	N = 55 0.4 (0.31–0.56)	N = 11 0.5 (0.37–0.7)	N = 44 0.4 (0.3–0.56)	0.438
AST (median, IQR)	N = 56 131.5 (63.3–268.8)	N = 12 188 (86–393)	N = 44 123 (53.2–261)	0.396
ALT (median, IQR)	N = 56 58 (30.7–114.5)	N = 12 77.5 (46.7–130.3)	N = 44 53.5 (29.3–114.5)	0.632

APACHE, Acute Physiology and Chronic Health Evaluation; ICU, intensive care unit; IQR, interquartile range; WBC, white blood cell; PLT, platelet count; CRP, C-reactive protein; CK, creatine kinase; LDH, lactic dehydrogenase; aPTT, activated partial thromboplastin time; AST, aspartate aminotransferase; ALT, alanine aminotransferase.

**Table 2:** The relationship between viral RNA load and clinical features of patients with SFTS.

Variables	Association with Viral Load		Values Corresponding to the Indicated Number of RNA Copies/mL		
	ρ *	*p* **	<10,000 (*n* = 35)	≥10,000 (*n* = 21)	*p* ***
**Number of patients with tick bite (%)**	0.004	0.977	12 (34.3%)	7 (33.3%)	0.992
**WBC**	−0.062	0.648	1980 (1210–2740)	1810 (1370–2250)	0.407
**Neutrophils**	0.282+	0.040	59.4 (51.7–69.7)	66.7 (61–72)	0.531
**PLT**	−0.079	0.559	82 (59–100)	66 (45–101)	0.524
**LDH**	−0.059	0.741	966 (652–2293)	986 (528–1361)	0.472
**CK**	−0.034	0.839	640 (168–1445)	470 (219–1084)	0.474
**Serum bicarbonate concentration (mmol/L)**	−0.112	0.554	23 (19–25)	22 (20–24)	0.581
**Serum creatinine (mg/dl)**	0.1	0.456	0.84 (0.7–1.2)	1.03 (0.7–1.3)	0.295
**Direct serum bilirubin**	0.137	0.314	0.4 (0.3–0.6)	0.4 (0.37–0.55)	0.378
**Aspartate aminotransferase**	0.066	0.624	124(50–269)	145 (67–269)	0.365
**Alanine** **aminotransferase**	0.013	0.926	64 (30–103)	53 (34–136)	0.594
**Alkaline phosphate**	0.089	0.541	69 (60–93)	75 (58–112)	0.439
**CRP**	0.604 +	0.024	0.14 (0.04–0.6)	1.5 (0.9–2.0)	0.370
**aPTT**	0.321 +	0.028	37.3 (34–45)	45 (37–54)	0.513
**APACH II score**	0.138	0.340	10 (8–13)	11 (8–14)	0.237
**Fatality**	0.494 +	<0.001	2 (5.7%)	10 (47.6%)	<0.001
**ICU admission**	0.390 +	0.004	8 (22.9%)	13 (61.9%)	0.003
**Complications**	0.116	0.393	17 (48.6%)	13 (61.9%)	0.389

+ <0.05. * Spearman’s rho test; ** Mann–Whitney *U* test; *** Fisher’s exact test.

**Table 3:** Logistic regression analysis between the survival and non-survival groups.

Variables	Univariate			Multivariate		
	OR	95% CI	*p*	OR	95% CI	*p*
SFTS copy (cut off 10,000)	0.067	0.013–0.352	0.001	38.298	1.583–926.593	0.025
Underlying disease	0.318	0.076–1.340	0.12	1.425	0.183–11.117	0.736
Initial APACHE II score	1.24	1.048–1.466	0.012	1.306	1.031–1.655	0.027
Days from symptom onset to admission	1.24	0.12–13.15	0.857	1.104	0.759–1.606	0.605
Age	1.115	1.028-1.211	0.009	1.034	0.920-1.162	0.573

## Data Availability

Data and materials are available upon request to the corresponding author.

## Supplementary Materials

The following are available online at https://www.mdpi.com/article/10.3390/v14050881/s1, Table S1, Oligonucleotide primers and probes for SFTSV-specific real-time PCR.
