# Supplementary material for: Viral Load as a Factor Affecting the Fatality of Patients Suffering from Severe Fever with Thrombocytopenia Syndrome
Source: Viruses. 2022 Apr 23;14(5):881. doi: 10.3390/v14050881 (PMC9144926; doi:10.3390/v14050881)
Supplement: Supplementary file 1 [file viruses-14-00881-s001.zip › viruses-1643508-supplementary.pdf]

Supplementary Table S1. Oligonucleotide primers and probes for SFTSV-specific real-time PCR.

Virus	Primer Name	Primer Sequence (5′→3′)	PCR Product (bp)	Reference
**SFTSV**	SFTS-SQ-F	ACCTCTTTGACCCTGAGTTWGACA	120	Zhang et al., 2012
	SFTS-SQ-R	CTGAAGGAGACAGGTGGAGATGA		
	SFTS-SQ-P	FAM-TGCCTTGACGATCTTA-NFQ-MGB		
